# An open-label prospective randomized multicenter study of intensive versus weekly granulocyte and monocyte apheresis in active crohn’s disease

**DOI:** 10.1186/s12876-015-0390-3

**Published:** 2015-11-19

**Authors:** Naoki Yoshimura, Yoko Yokoyama, Katsuyoshi Matsuoka, Hiroki Takahashi, Ryuichi Iwakiri, Takayuki Yamamoto, Tomoo Nakagawa, Takumi Fukuchi, Satoshi Motoya, Reiko Kunisaki, Shingo Kato, Fumihito Hirai, Yoh Ishiguro, Satoshi Tanida, Sakiko Hiraoka, Keiichi Mitsuyama, Shunji Ishihara, Shinji Tanaka, Michiro Otaka, Taro Osada, Takashi Kagaya, Yasuo Suzuki, Hiroshi Nakase, Hiroyuki Hanai, Kenji Watanabe, Nobuhito Kashiwagi, Toshifumi Hibi

**Affiliations:** Department of internal medicine, Division of IBD, Tokyo Yamate Medical Centre, Tokyo, Japan; Division of Internal Medicine, Department of Inflammatory Bowel Disease, Hyogo College of Medicine, Hyogo, Japan; Department of Gastroenterology and Hepatology, Tokyo Medical and Dental University, Tokyo, Japan; Department of General Medicine, National Hospital Organization, Sendai Medical Centre, Miyagi, Japan; Division of Gastroenterology, Department of Internal Medicine, Saga Medical School, Saga, Japan; Inflammatory Bowel Disease Centre, Yokkaichi Hazu Medical Centre, Mie, Japan; Department of Gastroenterology and Hepatology, Chiba University Hospital, Chiba, Japan; Department of Gastroenterology and Hepatology, Osakafu Saiseikai Nakatsu Hospital, Osaka, Japan; IBD Center, Sapporo Kosei General Hospital, Hokkaido, Japan; Inflammatory Bowel Centre, Yokohama City University Medical Centre, Kanagawa, Japan; Division of Gastroenterology and Hepatology, Department of Internal Medicine, Saitama Medical Centre, Saitama Medical University, Saitama, Japan; Department of Gastroenterology Fukuoka University Chikushi Hospital, Fukuoka, Japan; Department of Gastroenterology and Hematology, Hirosaki National Hospital, Aomori, Japan; Department of Gastroenterology and Metabolism, Nagoya City University, Graduate School of Medical Sciences, Nagoya, Japan; Department of Gastroenterology and Hepatology, Dentistry and Pharmaceutical Sciences, Okayama University Graduate School of Medicine, Okayama, Japan; Division of Gastroenterology, Department of Medicine, Inflammatory Bowel Disease Centre, Kurume University School of Medicine, Fukuoka, Japan; Department of Internal Medicine II, Faculty of Medicine, Shimane University, Izumo, Japan; Department of Endoscopy, Hiroshima University Hospital, Hiroshima, Japan; Division of Gastroenterology, Kobari General Hospital & Juntendo University, Chiba, Japan; Department of Gastroenterology, Juntendo University School of Medicine, Tokyo, Japan; Department of Gastroenterology, Kanazawa University Hospital, Ishikawa, Japan; Internal Medicine, Toho University Sakura Medical Centre, Chiba, Japan; Department of Gastroenterology and Endoscopic Medicine, Kyoto University Hospital, Kyoto, Japan; Centre for Gastroenterology and Inflammatory Bowel Disease Research, Hamamatsu South Hospital, Shizuoka, Japan; Department of Gastroenterology, Osaka City University Graduate School of Medicine, Osaka, Japan; Research division, JIMRO Co., Ltd, Gunma, Japan; Kitasato Institute Hospital, Centre for Advanced IBD Research and Treatment, Kitasato University, 108-8642 Minato-ku, Tokyo, Japan

**Keywords:** Crohn’s disease, Prospective study, Granulocyte and monocyte adsorptive apheresis, Intensive therapy, Leucocyte count, Erythrocyte sedimentation rate, C-reactive protein

## Abstract

**Background:**

Granulocyte and monocyte adsorptive apheresis (GMA) has shown efficacy in patients with active Crohn’s disease (CD). However, with routine weekly therapy, it may take several weeks to achieve remission. This study was performed to assess clinical efficacy and safety of intensive GMA in patients with active CD.

**Methods:**

In an open-label, prospective, randomized multicentre setting, 104 patients with CD activity index (CDAI) of 200 to 450 received intensive GMA, at two sessions per week (*n* = 55) or one session per week (*n* = 49). Clinical remission was defined as a CDAI score <150. Patients in each arm could receive up to 10 GMA sessions. However, GMA treatment could be discontinued when CDAI decreased to <150 (clinical remission level).

**Results:**

Of the 104 patients, 99 were available for efficacy evaluation as per protocol, 45 in the weekly GMA group, and 54 in the intensive GMA group. Remission was achieved in 16 of 45 patients (35.6 %) in the weekly GMA and in 19 of 54 (35.2 %) in the intensive GMA (NS). Further, the mean time to remission was 35.4 ± 5.3 days in the weekly GMA and 21.7 ± 2.7 days in the intensive GMA (*P* = 0.0373). Elevated leucocytes and erythrocyte sedimentation rate were significantly improved by intensive GMA, from 8005/μL to 6950/μL (*P* = 0.0461) and from 54.5 mm/hr to 30.0 mm/hr (*P* = 0.0059), respectively. In both arms, GMA was well tolerated and was without safety concern.

**Conclusions:**

In this study, with respect to remission rate, intensive GMA was not superior to weekly GMA, but the time to remission was significantly shorter in the former without increasing the incidence of side effects. UMIN registration # 000003666.

**Electronic supplementary material:**

The online version of this article (doi:10.1186/s12876-015-0390-3) contains supplementary material, which is available to authorized users.

## Background

Biologics, such as anti-tumour necrosis factor (TNF)-α antibodies or conventional medications with 5-aminosalicylic acid, prednisolone, and immunomodulators like azathioprine or 6-mercaptopurine are being used to treat patients with active Crohn’s disease (CD) [1–6]. However, these drugs have often been associated with adverse side effects that add to disease complexity [7–11]. Further, the anti-TNF infliximab is effective for induction and maintaining remission in patients with CD [1, 12, 13], but more than 50 % of the patients may show loss of response to this biologic over a 2 year period [14]. Therefore, there is a need for effective and well-tolerated treatment options for CD patients. Granulocyte and monocyte adsorptive apheresis (GMA) is an extracorporeal therapy that is performed with the Adacolumn® (JIMRO, Takasaki, Japan). GMA selectively adsorbs elevated/activated granulocytes and monocytes, and very few lymphocytes from the peripheral blood [15]. A number of studies have reported on the therapeutic efficacy of GMA in patients with CD, ulcerative colitis (UC) and generalized pustular psoriasis [16–21]. Additionally, GMA has shown efficacy in patients with CD refractory to conventional medication [22, 23].

However, with the routinely applied weekly GMA, it may take several weeks to see the treatment efficacy, because the Japan healthcare insurance covers only the one GMA session per week regimen. With this in mind, we hypothesized that an intensive course of GMA should produce a more rapid efficacy compared with weekly GMA as reported by Sakuraba, et al. for patients with UC [24]. This prospective, randomized, multicenter study was undertaken to evaluate the safety, efficacy, and time to remission for intensive GMA, alongside the routine weekly GMA in patients with active, mild-to-moderate CD.

## Methods

### Study setting

This was an open-label, prospective, randomized, multicenter study aimed at evaluating the clinical efficacy, safety, and the appropriate treatment schedule for GMA in patients with mild-to-moderately active CD. The study was conducted at 31 medical institutions from June 2010 to July 2014. Among the 31 study centers, 22 were academic, university institutes, 7 were municipal hospital centers, and 2 were private community clinics. All involved centers had adequate experience to undertake GMA therapy for patients with inflammatory bowel disease (IBD) (Additional file 1).

### Patients

Male or non-pregnant female with a definitive diagnosis of CD (12–75 years of age) were eligible if they had their first CD episode or had relapsed with mild-to- moderately active CD. The severity of CD was determined by the Crohn’s disease activity index (CDAI) according to Becktel et al. [25]. Patients who had a CDAI score of 200 to 450 were classified as having mild-to-moderately active CD and were eligible. Disease location was to be in the colon or in the ileum and the colon (ileocolic). Patients with granulocytopaenia (neutrophil count <2000 per μl), serious heart or kidney malfunction, coagulation disorder, or had infection were to be excluded. Further, patients who had started a corticosteroid within one week, adalimumab within 2 weeks or infliximab within 6 weeks were excluded. Oral maintenance aminosalicylate was allowed if it had been given at a stable dose for at least 2 weeks before entry. This was at least 8 weeks for azathioprine/6-mercaptopurine. Concomitant medications for diseases other than CD, which did not violate the protocol inclusion criteria were allowed.

### Allocations of patients to the study arms

After screening and enrollment for this study, patients were randomly assigned to receive weekly GMA or receive intensive GMA, at two sessions per week in a 1:1 ratio. Randomization was done by a central computer-generated randomization scheme that assigned the eligible patient to a study centre.

### GMA procedures

GMA treatment was done as previously described [16]. Briefly, the Adacolumns® and blood circuit lines were primed with sterile saline to remove air bubbles from the column void volume and flow lines. A second priming of the system was done with heparinized saline. Blood access was through the antecubital vein in one arm and from the column outflow, blood returned to the patient via the antecubital vein in the contralateral arm. The duration of one GMA session was 60 min at a flow rate of 30 ml/min. Treatment was carried out partly in an outpatient setting and partly in an inpatient setting. Patients in the weekly GMA arm received one GMA session per week and those in the intensive GMA arm received two treatments sessions per week. The maximum number of GMA sessions allowed was 10. However, when a patient achieved remission (CDAI <150), GMA could be discontinued. The treatment and observation time was 77 days in the weekly GMA and 42 days in the intensive GMA.

### Assessment of the treatment outcomes

With respect to the CD activity level, each patient was evaluated by determining the CDAI score at screening, baseline, and before each GMA session. The study primary end point was the rate of clinical remission defined as CDAI <150. The secondary end point was the time to remission, a key measurement for comparison of intensive GMA with weekly GMA. Evaluation of time to remission was according to a life-table analysis by using the Kaplan-Meier estimator graphs.

### Ethical considerations

In Japan, GMA with the Adacolumn® is an officially approved treatment option for patients with IBD. However, before initiating the GMA therapy, our study protocol and patients’ informed consent forms were reviewed and approved by the Institutional Review Board at each study centre. Patients agreed to participate in this study after being informed of the study purpose, actions of GMA and the nature of the procedures involved. In case of an under-age patient, consent from one of the patient’s parents was sought. Written informed consent was obtained from all patients. The study was conducted with strict adherence to the Helsinki Declaration.

### Statistical analysis

Data are presented as the mean ± SEM values or the median with inter-quartile range. For statistical analysis, data were processed by using a JMP software (version 10, SAS Institute, Cary, NC). Categorical data were analyzed by Fisher’s exact test, while continuous data were evaluated by using the Wilcoxon-Mann–Whitney test or as indicated otherwise in figure and table legends. *P* < 0.05 was considered statistically significant. The sample size was determined as follows. This trial was designed as a superiority study. To show a statistical difference in the remission rate between an assumption of a 30 % efficacy in the weekly GMA and a 50 % efficacy in the intensive GMA, with a first-kind error of 5 % and power of an 80 %, a sample size of 93 patients per group in the per protocol population was estimated. With approximately 5 % withdrawal, a total of 100 patients had to be included in each arm. However, when the patient number in each arm reached around 50, an interim analysis was done, which showed similar remission rates in the two arms. We then decided to stop further patients enrollment and carried out data analyses based on 104 patients.

## Results

### Patient randomization and demography

**Fig. 1:**
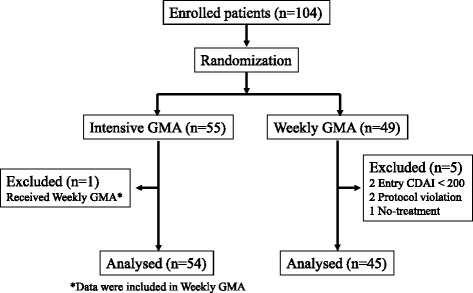
Patient disposition. CDAI = Crohn’s disease activity index; GMA = granulocyte and monocyte apheresis

A total of 104 patients who were registered after screening were randomized to the weekly GMA arm (*n* = 49) or to the intensive GMA arm (*n* = 55). Five patients in the weekly GMA could not be included in the efficacy analysis, two had not reached CDAI score ≥200 at screening, one patient was not available for GMA therapy and the remaining two patients were found to have received GMA prior to assignment. Additionally, one patient who was assigned to the intensive GMA arm had received weekly GMA, this patient was included in the weekly GMA arm during the analysis of the treatment outcome (Fig. 1). Therefore, a total of 99 cases (45 in the weekly GMA and 54 in the intensive GMA) were available for efficacy evaluations as per protocol. There was no significant difference between the two groups with respect to patients’ baseline demographic variables including gender, age, CD duration, inpatient, outpatient, CDAI score and disease location (Table 1). Concomitant conventional pharmacological agents used by these patients included sulphasalazine, 5-aminosalicylic acid, prednisolone, azathioprine, and 6-mercaptopurine. There was no significant difference between the two groups with respect to concomitant medications.

**Table 1:** Baseline demographic variables of the 99 patients with active Crohn’s disease who were assigned to intensive GMA or to weekly GMA in this study

Demography	Intensive GMA	Weekly GMA	*P* value
	(*n* = 54)	(*n* = 45)	
Male gender - n (%)	34 (63.0)	24 (53.3)	0.4133
Age, year	31.5 [24.8–40.5]	30.0 [23.5–43.0]	0.8965
Duration of disease, month	36.0 [5.5–138.0]	44.0 [11.5–93.0]	0.7081
Hospitalization - n (%)	27 (50.0)	22 (48.9)	1.0000
CDAI	260 [235–310]	259 [224–320]	0.9300
Disease location - n (%)			
Colon	23 (42.6)	17 (37.8)	0.6838
Ileum and colon	31 (57.4)	28 (62.2)	0.6838
Surgical history – n (%)	13 (24.1)	15 (33.3)	0.3724
Previous biologics treatment - n (%)	10 (18.5)	10 (22.2)	0.8022
Concomitant medication - n (%)			
5-Aminosalicylates	46 (85.2)	37 (82.2)	0.7865
Corticosteroids	8 (14.8)	8 (17.8)	0.7865
Azathioprine	7 (13.0)	9 (20.0)	0.4157
6-Mercaptopurine	3 (5.6)	2 (4.4)	1.0000
Nutrition therapy – n (%)	37 (68.5)	31 (68.9)	1.0000

Continuous variables are presented as the median [interquartile range] and were compared by the Wilcoxon-Mann–Whitney test. Categorical variables are presented as patient’s number (%) and were compared by the Fisher’s exact test. CDAI, Chrohn’s disease activity index; GMA, granulocyte and monocyte adsorptive apheresis

### The primary efficacy outcome

As stated above, 45 of 49 patients randomized to the weekly GMA arm and 54 of 55 patients randomized to the intensive GMA arm were available for efficacy assessment as per protocol. During the 77 days of the study period in the weekly GMA arm, 16 of 45 patients (35.6 %) achieved clinical remission. Likewise, during the 42 days of the study period in the intensive GMA arm, 19 of 54 patients (35.2 %) achieved clinical remission, not significantly different from the weekly GMA arm.

### The secondary efficacy outcome

**Fig. 2:**
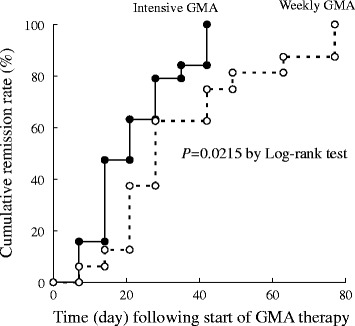
The Kaplan-Meier survival graphs showing cumulative remission rates for patients who achieved clinical remission. In the weekly GMA arm (dotted line), patients received one session per week, while in the intensive arm (solid line), patients received two GMA sessions per week. The mean time to remission for the 16 patients in the weekly GMA arm who achieved clinical remission was 35.4 ± 5.3 days vs 21.7 ± 2.7 days in the 19 patients of the intensive GMA arm who achieved clinical remission, *P* = 0.0373 by Wilcoxon-Mann–Whitney test. GMA = granulocyte and monocyte adsorptive apheresis

In Fig. 2, the Kaplan-Meier estimator graphs show the cumulative remission rate in the weekly and the intensive GMA arms. The mean time to remission among the 16 patients in the weekly GMA who achieved clinical remission was 35.4 ± 5.3 days, while the mean time to remission in the 19 patients of the intensive GMA arm who achieved remission was 21.7 ± 2.7 days (*P* = 0.0373). Therefore, in CD patients, intensive GMA was associated with significantly more rapid remission as compared with the routinely applied weekly GMA.

### Changes in leucocyte count, and inflammation markers

In spite of randomized assignment of enrolled patients, baseline leucocyte count (normal range: 4000–9000/μL), erythrocyte sedimentation rate (ESR) (normal range: < 10 mm/hr (male); < 15 mm/hr (female)) and C-reactive protein (CRP) (normal range: < 0.3 mg/dL) concentration were found to be significantly higher in the intensive GMA group compared with the weekly GMA group, 8005/μL vs 7100/μL (*P* = 0.0273), 54.5 mm/hr vs 24.5 mm/hr (*P* = 0.0146) and 1.90 mg/dL vs 0.90 mg/dL (*P* = 0.0478), respectively. However, only in the intensive GMA group, leucocyte count (*P* = 0.0461) and ESR (*P* = 0.0059) were significantly reduced. Likewise, CRP level had decreased from 1.90 mg/dL to 0.80 mg/dL (*P* = 0.0941). In the weekly GMA, CRP level was significantly (*P* = 0.0277) decreased from 0.90 mg/dL at baseline to 0.35 mg/dL (*P* = 0.0277), but, leucocyte count and ESR level were not significantly affected (Table 2).

**Table 2:** Changes in leucocyte count, erythrocyte sedimentation rate (ESR) and C-reactive protein (CRP) following intensive and weekly GMA in Crohn’s disease patients

Measurement	Time point	Intensive GMA	Weekly GMA	*P* value*, intensive vs weekly
Leucocyte (/μL)	Baseline	8005 [6290–9378]	7100 [5340–7915]	0.0273
	Post GMA	6950 [5400–8775]	7160 [5338–8465]	0.8601
*P* value**				
Baseline vs Post GMA		0.0461	0.6260	
ESR (mm/hr)	Baseline	54.5 [37.0–68.0]	24.5 [9.7–52.5]	0.014
	Post GMA	30.0 [10.5–51.5]	26.0 [7.0–47.0]	0.5480
*P* value**				
Baseline vs Post GMA		0.0059	0.6553	
CRP (mg/dL)	Baseline	1.90 [0.60–4.05]	0.90 [0.40–2.62]	0.0478
	Post GMA	0.80 [0.12–2.30]	0.35 [0.195–1.27]	0.4464
*P* value**				
Baseline vs Post GMA		0.0941	0.0277	

Data are represented as the median [interquartile range]

*Compared by Wilcoxon-Mann–Whitney test

**Compared by Wilcoxon-Signed-Rank test. GMA, granulocyte and monocyte adsorptive apheresis. For ESR, the number of patients at baseline in the intensive and the weekly GMA arms were 22 and 24, respectively. Normal range: leucocyte 4000–9000/μL, CRP < 0.3 mg/dL, ESR < 10 mm/hr (male); < 15 mm/hr (female)

### Treatment safety and tolerability

No device-related serious adverse event or unexpected adverse event was observed in either group. Adverse events were consistent with those generally observed during extracorporeal procedures including transient headache, dizziness, flushing, and pyrexia (Table 3). A total of 22 patients experienced none serious adverse events in the weekly GMA (*n* = 11) and the intensive GMA (*n* = 11) arms. In 5 of 11 patients in the weekly GMA and 6 of 11 patients in the intensive GMA, the adverse events were considered to be likely treatment related. No episode of opportunistic infection was observed in either group, suggesting that therapeutic depletion of large numbers of leucocytes by GMA might not lead to a weakened immune function. Further, both intensive and weekly GMA treatments were well tolerated by patients and there were no technical problems.

**Table 3:** Reported adverse events during the study

System organ class and preferred term	Intensive GMA (*N* = 54)	Weekly GMA (*N* = 45)	Total
Nervous system disorder			8 (8.1)
Headache	2 (3.7)	5 (11.1)	
Dizziness	1 (1.9)	0 (0.0)	
General disorders and administration site condition			4 (4.0)
Pyrexia	2 (3.7)	1 (2.2)	
Chest pain	1 (1.9)	0 (0.0)	
Gastrointestinal disorders			2 (2.0)
Abdominal pain	0 (0.0)	1 (2.2)	
Diarrhea	0 (0.0)	1 (2.2)	
Cardiac disorders			2 (2.0)
Palpitations	1 (1.9)	0 (0.0)	
Coldness/Bradycaridia	1 (1.9)	0 (0.0)	
Musculoskeletal connective tissue disorders			2 (2.0)
Arthralgia	0 (0.0)	1 (2.2)	
Myalgia	1 (1.9)	0 (0.0)	
Vascular disorders			2 (2.0)
Hot flashes	0 (0.0)	1 (2.2)	
Hypotension	1 (1.9)	0 (0.0)	
Blood and lymphatic system disorders			1 (1.0)
Leukopenia	0 (0.0)	1 (2.2)	
Ear and labyrinth disorders			1 (1.0)
Ear fullness	1 (1.9)	0 (0.0)	
Total	11 (20.4)	11 (24.4)	22 (22.2)

Values are the patient’s number (%)

## Discussion

It has been reported that an intensive, two GMA session per week produced a higher remission rate and in a shorter time as compared with one GMA session per week in patients with active UC [24]. In this study, we found that intensive GMA as two sessions per week, up to 10 sessions and weekly GMA, up to 10 sessions were associated with similar remission rates in patients with active CD, but time to remission was significantly better with the former as compared with the latter. The mean time to remission was 5 weeks in the weekly GMA vs 3 weeks in the intensive GMA. The obvious difference between the two treatment frequencies appears to be shortening of morbidity time by intensive GMA.

Dignass et al. compared 5 and 10 GMA apheresis treatments in steroid-refractory or steroid–dependent UC patients, and reported that 5 GMA sessions were not inferior to 10 sessions [26]. In their study protocol, patients received 5 GMA treatments, once a week over 5 consecutive weeks, or 10 treatments, twice weekly for the first 2 weeks followed by treatments once a week for the following 6 consecutive weeks. Comparing our results with theirs, it might be important to conduct more GMA treatments in a week to obtain a shorter time to remission.

Leucocyte counts, ESR and CRP levels are known as biomarkers of inflammation that correlate well with disease activity in patients with IBD as well as in patients with other inflammatory diseases [27, 28]. In this study, intensive GMA (but not weekly GMA) was associated with a significant decrease in the elevated leucocyte count, and ESR, reflecting amelioration of the inflammation profile in the treated patients. Delayed apoptosis (prolonged survival) and abnormal function of neutrophils have been reported in patients with CD [29, 30]. Neutrophils apoptosis is known to be an important event in the maintenance of immune homeostasis [31]. However, endotoxin may be detectable during mucosal inflammation, which can activate neutrophils [32]. Additionally, an increased levels of pro-inflammatory cytokines including granulocyte macrophage colony-stimulating factor and macrophage migration inhibitory factor are seen in patients with active IBD [33, 34]. These mediators together with corticosteroids, which are given to most patients with active IBD inhibit neutrophil apoptosis [35, 36]. These understandings support the notion that in patients with active IBD, selective depletion of myeloid lineage leucocytes (neutrophils and monocytes/macrophages) may induce disease amelioration.

Further, Ishihara, et al. [37] reported that GMA with the Adacolumn® induced neutrophil apoptosis during passage of blood through the GMA column and exposure of neutrophils to reactive oxygen species, which are generated in the column. The authors reported amelioration of induced colitis in animal models of IBD [37]. Similarly, the authors suggested that neutrophil apoptosis was induced via phagocytosis induced cell death reaction in the column, and the apoptotic neutrophils can be taken up by phagocytes including dendritic cells and macrophages [38]. Finally, regulatory B cells (Bregs) expanded in the model, and ameliorated colonic inflammation [37]. Their results suggested that the Adacolumn® not only selectively removes neutrophils and monocytes from the circulation but also indirectly promotes expansion of Bregs, which are involved in maintaining regulatory T cells (Tregs) [39]. In clinical settings, the Adacolumn® has been associated with expansion of Tregs, an increase in interleukin-10 level and a decrease of anti-nuclear antibodies titer [40–42].

We were aware several limitations of this study. Firstly, this was an open-label study and we did not use sham columns in the weekly GMA group. More frequent procedures in the intensive GMA group may have affected the results. Secondary, we evaluated the patients by the CDAI, which is a subjective measurement. We did not include objective inflammatory markers such as fecal calprotectin as an inclusion criterion. Consequently, some patients might have had minimal intestinal inflammation despite their gastrointestinal symptoms at entry. Thirdly, we did not perform any imaging evaluation such as endoscopy, ultrasonography, and magnetic resonance enterography in this study.

## Conclusion

In this study, applying intensive GMA to deplete elevated and activated myeloid lineage leucocytes in patients with active CD resulted in a more rapid clinical remission than weekly GMA, but without inducing an increased remission rate. Therefore, in this study, with respect to remission rate, intensive GMA was not superior to weekly GMA, a shortening of the morbidity time was the only obvious and clinically relevant benefit of intensive GMA. Further, GMA is generally favored by patients for its safety feature as well as for being a non-pharmacologic treatment intervention.

## Additional file

Additional file 1:
**CONSORT 2010 checklist.** (DOC 217 kb)

## Abbreviations

CD: Crohn’s disease
CDAI: Crohn’s disease activity index
CRP: C-reactive protein
ESR: Erythrocyte sedimentation rate
GMA: Granulocyte and monocyte apheresis
IBD: Inflammatory bowel disease

## References

[1] Colombel JF, Loftus EV, Tremaine WJ, Egan LJ, Harmsen WS, Schleck CD (2004). The safety profile of infliximab in patients with Crohn’s disease: the Mayo clinic experience in 500 patients. Gastroenterology.

[2] Munkholm P, Langholz E, Davidsen M, Binder V (1994). Frequency of glucocorticoid resistance and dependency in Crohn’s disease. Gut.

[3] Otley A, Steinhart AH (2005). Budesonide for induction of remission in Crohn’s disease. Cochrane Database Syst Rev.

[4] Scott FI, Osterman MT (2013). Medical management of crohn disease. Clin Colon Rectal Surg.

[5] Roberts RL, Barclay ML (2012). Current relevance of pharmacogenetics in immunomodulation treatment for Crohn’s disease. J Gastroenterol Hepatol.

[6] Levesque BG, Loftus EV (2012). Initiating azathioprine for Crohn’s disease. Clin Gastroenterol Hepatol.

[7] Sokumbi O1, Wetter DA, Makol A, Warrington KJ (2012). Vasculitis associated with tumor necrosis factor-α inhibitors. Mayo Clin Proc.

[8] Beigel F, Jürgens M, Filik L, Bader L, Lück C, Göke B (2009). Severe Legionella pneumophila pneumonia following infliximab therapy in a patient with Crohn’s disease. Inflamm Bowel Dis.

[9] Dasari BV, McBrearty A, Gardiner K (2012). Immunosuppression in patients with Crohn’s disease and neoplasia: an ongoing clinical dilemma. Dis Colon Rectum.

[10] Das KM, Eastwood MA, McManus JP, Sircus W (1973). Adverse reactions during salicylazosulfapyridine therapy and the relation with drug metabolism and acetylator phenotype. N Engl J Med.

[11] Marinaki AM, Ansari A, Duley JA, Arenas M, Sumi S, Lewis CM (2004). Adverse drug reactions to azathioprine therapy are associated with polymorphism in the gene encoding inosine triphosphate pyrophosphatase (ITPase). Pharmacogenetics.

[12] Yoshida K, Fukunaga K, Ikeuchi H, Kamikozuru K, Hida N, Ohda Y (2012). Scheduled infliximab monotherapy to prevent recurrence of Crohn’s disease following ileocolic or ileal resection: A 3-year prospective randomized open trial. Inflamm Bowel Dis.

[13] Alzafiri R, Holcroft CA, Malolepszy P, Cohen A, Szilagyi A (2011). Infliximab therapy for moderately severe Crohn’s disease and ulcerative colitis: a retrospective comparison over 6 years. Clin Exp Gastroenterol.

[14] Ma C, Huang V, Fedorak DK, Kroeker KI, Dieleman LA, Halloran BP (2014). Crohn’s disease outpatients treated with adalimumab have an earlier secondary loss of response and requirement for dose escalation compared to infliximab: A real life cohort study. J Crohns Colitis.

[15] Saniabadi AR, Hanai H, Takeuchi K, Sawada K, Fukunaga K, Lofberg R (2003). Adacolumn, an adsorptive carrier based granulocyte and monocyte apheresis device for the treatment of inflammatory and refractory diseases associated with leukocytes. Ther Apher Dial.

[16] Shimoyama T, Sawada K, Hiwatashi N (2001). Safety and efficacy of granulocyte and monocyte adsorption apheresis in patients with active ulcerative colitis: A multicenter study. J Clin Apher.

[17] Fukuda Y, Matsui T, Suzuki Y (2004). Adsorptive granulocyte and monocyte apheresis for refractory Crohn’s disease: an open multicenter prospective study. J Gastroenterol.

[18] Fukuchi T, Nakase H, Ubukata S, Matsuura M, Yoshino T, Toyonaga T (2014). Therapeutic effect of intensive granulocyte and monocyte adsorption apheresis combined with thiopurines for steroid- and biologics-naïve Japanese patients with early-diagnosed Crohn’s disease. BMC Gastroenterol.

[19] Martín de Carpi J, Vilar P, Prieto G, García Novo MD, Ribes C, Varea V (2008). Safety and efficacy of granulocyte and monocyte adsorption apheresis in paediatric inflammatory bowel disease: a prospective pilot study. J Pediatr Gastroenterol Nutr.

[20] Giampaolo B, Giuseppe P, Michele B, Alessandro M, Fabrizio S, Alfonso C (2006). Treatment of active steroid-refractory inflammatory bowel diseases with granulocytapheresis: Our experience with a prospective study. World J Gastroenterol.

[21] Ikeda S, Takahashi H, Suga Y, Eto H, Etoh T, Okuma K (2013). Therapeutic depletion of myeloid lineage leukocytes in patients with generalized pustular psoriasis indicates a major role for neutrophils in the immunopathogenesis of psoriasis. J Am Acad Dermatol.

[22] Sacco R, Romano A, Mazzoni A, Bertini M, Federici G, Metrangolo S (2013). Granulocytapheresis in steroid-dependent and steroid-resistant patients with inflammatory bowel disease: A prospective observational study. J Crohns Colitis.

[23] Lindberg A, Eberhardson M, Karlsson M, Karlén P (2010). Long-term follow-up with Granulocyte and Monocyte Apheresis re-treatment in patients with chronically active inflammatory bowel disease. BMC Gastroenterol.

[24] Sakuraba A, Motoya S, Watanabe K, Nishishita M, Kanke K, Matsui T (2009). An open-label prospective randomized multicenter study shows very rapid remission of ulcerative colitis by intensive granulocyte and monocyte adsorptive apheresis as compared with routine weekly treatment. Am J Gastroenterol.

[25] Best WR, Becktel JM, Singleton JW (1979). Rederived values of the eight coefficients of the Crohn’s Disease Activity Index (CDAI). Gastroenterology.

[26] Dignass AU, Eriksson A, Kilander A, Pukitis A, Rhodes JM, Vavricka S (2010). Clinical trial: five or ten cycles of granulocyte-monocyte apheresis show equivalent efficacy and safety in ulcerative colitis. Aliment Pharmacol Ther.

[27] Charron M (2003). Inflammatory bowel disease activity assessment with biologic markers and 99mTc-WBC scintigraphy: Are there different trends in ileitis versus colitis?. J Nucl Med.

[28] Vermeire S, Van Assche G, Rutgeerts P (2006). Laboratory markers in IBD: useful, magic, or unnecessary toys?. Gut.

[29] Somasundaram R, Nuij VJ, van der Woude CJ, Kuipers EJ, Peppelenbosch MP, Fuhler GM (2013). Peripheral neutrophil functions and cell signalling in Crohn’s disease. PLoS One.

[30] Brannigan AE, O’Connell PR, Hurley H, O’Neill A, Brady HR, Fitzpatrick JM (2000). Neutrophil apoptosis is delayed in patients with inflammatory bowel disease. Shock.

[31] Sookhai S, Wang JH, McCourt M, O’Connell D, Redmond HP (1999). Dopamine induces neutrophil apoptosis through a dopamine D-1 receptor independent mechanism. Surgery.

[32] Pastor Rojo O, López San Román A, Albéniz Arbizu E, de la Hera Martínez A, Ripoll Sevillano E (2007). Serum lipopolysaccharide-binding protein in endotoxemic patients with inflammatory bowel disease. Inflamm Bowel Dis.

[33] Ina K, Kusugami K, Hosokawa T, Imada A, Shimizu T, Yamaguchi T (1999). Increased mucosal production of granulocyte colony-stimulating factor is related to a delay in neutrophil apoptosis in Inflammatory Bowel disease. J Gastroenterol Hepatol.

[34] Nishihira J, Mitsuyama K (2009). Overview of the role of macrophage migration inhibitory factor (MIF) in inflammatory bowel disease. Curr Pharm Des.

[35] Akgul C, Moulding DA, Edwards SW (2001). Molecular control of neutrophil apoptosis. FEBS Lett.

[36] Liles WC, Dale DC, Klebanoff SJ (1995). Glucocorticoids inhibit apoptosis of human neutrophils. Blood.

[37] Ansary MM, Ishihara S, Oka A, Kusunoki R, Oshima N, Yuki T (2014). Apoptotic cells ameliorate Chronic Intestinal Inflammation by Enhancing Regulatory B-cell Function. Inflamm Bowel Dis.

[38] Kennedy AD, DeLeo FR (2009). Neutrophil apoptosis and the resolution of infection. Immunol Res.

[39] Flores-Borja F, Bosma A, Ng D, Reddy V, Ehrenstein MR, Isenberg DA (2013). CD19+CD24hiCD38hi B cells maintain regulatory T cells while limiting TH1 and TH17 differentiation. Sci Transl Med.

[40] Cuadrado E, Alonso M, de Juan MD, Echaniz P, Arenas JI (2008). Regulatory T cells in patients with inflammatory bowel diseases treated with adacolumn granulocytapheresis. World J Gastroenterol.

[41] Yokoyama Y, Fukunaga K, Fukuda Y, Tozawa K, Kamikozuru K, Ohnishi K (2007). Demonstration of low-regulatory CD25High + CD4+ and high-pro-inflammatory CD28-CD4+ T-Cell subsets in patients with ulcerative colitis: modified by selective granulocyte and monocyte adsorption apheresis. Dig Dis Sci.

[42] Sono K, Yamada A, Yoshimatsu Y, Takada N, Suzuki Y (2012). Factors associated with the loss of response to infliximab in patients with Crohn’s disease. Cytokine.

